# Low HIV viral suppression rates following the intensive adherence counseling (IAC) program for children and adolescents with viral failure in public health facilities in Uganda

**DOI:** 10.1186/s12889-018-5964-x

**Published:** 2018-08-22

**Authors:** Esther Nasuuna, Joanita Kigozi, Lillian Babirye, Alex Muganzi, Nelson K. Sewankambo, Damalie Nakanjako

**Affiliations:** 1 Infectious Diseases Institute, Makerere University College of Health Sciences, P.O.Box 22418, Kampala, Uganda; 2 School of Medicine, Makerere University College of Health Sciences, P.O.Box 7072, Kampala, Uganda

**Keywords:** Detectable viral load, Viral load monitoring, Intensive adherence counseling (IAC), Adolescents, Pediatric, Adolescents, Uganda, Antiretroviral therapy

## Abstract

**Background:**

The UNAIDS 90–90-90 strategy clearly stipulates that 90% of all people on antiretroviral therapy (ART) should have a suppressed viral load. Intensified adherence counselling (IAC) was recently recommended by WHO to improve viral suppression among ART-treated paediatric and adolescent clients with virological failure. This paper describes the implementation and outcomes of IAC in the first year of implementation in a public ART program, to inform strategic interventions to reach the “third 90” among children.

**Methods:**

A retrospective chart review was conducted for all children aged 9 months to 19 years with HIV viral loads (VL) ≥ 1000 copies/ml at 15 public health facilities from June 2015–December 2016. Data on initial VL test results, IAC sessions, repeat VL test results, and ART regimen switch were abstracted and analysed for completion of IAC and viral suppression after IAC.

**Results:**

A total of 449 children had a detectable viral load above 1000 copies/ml, after an average of 3.5 years (SD 5.8) years of ART. 192 (43%) were 10–20 years of age, and 320 (71%) were receiving Nevirapine-based ART regimen. Out of 345 (77%) who completed the recommended three IAC sessions, 62 (23%) achieved viral suppression following IAC. The mean time from 1st to 3rd IAC session was 113 (SD 153) days and 172 (50%) of the children had completed the three sessions within 200 days.

**Conclusion:**

Suppression rates were low among ART-treated children with virological failure that completed the recommended three IAC sessions. As we move towards having 90% of ART-treated children and adolescents achieve and maintain viral suppression, there is need to re-evaluate the implementation of IAC among children and adolescents to consider both psychosocial and biological factors such as resistance testing for those with multiple detectable viral loads.

## Background

In 2014, UNAIDS released the 90–90-90 ambitious target to end the HIV epidemic globally by 2020. The third 90 clearly stipulates that 90% of all people receiving Antiretroviral Therapy (ART) should have HIV-RNA viral suppression [1]. Pediatric and adolescent clients (hereafter referred to as children) receiving ART, reportedly experience lower rates of viral suppression [2, 3] and would delay the achievement of this target if not given special consideration [4, 5]. It is important that children achieve protracted viral suppression during the lifelong ART to mitigate the exceptionally high mortality associated with unsuccessful HIV treatment [6]. Children have unique challenges in taking ART because of a multitude of factors such as total dependence on a caregiver, lack of disclosure, stigma, living with a non-biological caretaker and childish forgetfulness [7–9]. Moreover, treatment failure among children is under diagnosed and given little attention by HIV programs [4, 10].

It is generally believed that if adherence is addressed, clients receiving ART will achieve viral suppression, unless the ART regimen is failing [11]. The World Health Organization (WHO), basing on a systematic review that showed 70% of clients re-suppressed after adherence interventions, recommended adherence interventions for clients with high viral load [12, 13]. With over 61,000 children receiving ART in Uganda in 2016 [14], the government of Uganda adopted the WHO guidelines that each adult or child that had been receiving antiretroviral drugs for more than six months should have a viral load test done [15]. All individuals with HIV viral loads above 1000 copies would then receive IAC, which includes three sessions of monthly intensive adherence counselling sessions, followed by a repeat HIV viral load measurement. During the counselling sessions, children are supported to improve adherence to medication. After the repeat viral load test, children are supposed to either have a suppressed viral load (to continue with the first line regimen) or unsuppressed viral load (to get considered for a switch to a second line regimen) [13, 15].

The Infectious Diseases Institute (IDI) supports comprehensive HIV services in 15 Ugandan districts, providing ART for up to 12,392 children. The pediatric ART program services include support for children to stay adherent to medication in order to achieve and sustain viral suppression. Despite evidence that counselling, delivered at the start of ART, facilitates achievement of viral suppression [16]**,** there is limited literature about the success of IAC in a public health program setting among ART-treated children with unsuppressed viral load in Uganda. IDI pioneered the implementation of IAC in Uganda at her 15 HIV care sites. This paper describes the implementation and outcomes of IAC in the first year of implementation in a public ART program, among ART-treated children with viral loads above 1000 copies. Our results will inform strategic implementation of IAC programs to optimize outcomes of ART to achieve the “third 90” and have over 90% of ART-treated children with viral suppression.

## Methods

### Study design and population

This was a retrospective cohort study on all children aged 9 months to 19 years that had been enrolled into 15 HIV care programs. Data from all ART-treated children with a viral load above 1000 copies per ml, on a test done between June 2015 and December 2016, were analyzed. It is important to study children including the minors in the ART program in order to evaluate the outcomes and potential areas to improve IAC among all ART-treated children with unsuppressed viral loads.

### Program description

The government of Uganda adopted the WHO’s recommendations and the IAC program was implemented as stipulated by the national Ministry of Health (MoH) guidelines; to perform viral load measurement for all adults and children that have been receiving ART for at least six months. Individuals with viral load above 1000 copies/ml, undergo three adherence counselling sessions each one month apart, after which a viral load test is repeated. If the post-IAC viral load is suppressed, the clients continue with the current treatment and repeat the viral load after one year. If viral load is unsuppressed, clients are considered for a switch to a second line regimen after ensuring that adherence issues have been addressed. Resistance testing is only done for those who have suspected failure on a second line regimen before switch to a third regimen [13].

There are 2820 children registered in the 15 health facilities that were assessed. The health workers (nurses and adherence counsellors) initiate the IAC sessions; for children below five years, the sessions are run with the caregivers, for children six years to 15 years, the sessions are conducted together with the children and their caregivers and for the older adolescents, the sessions can be conducted for them without their caregivers. Expert clients (individuals on ART that are suppressing well and are volunteering in the ART clinic) also conduct the IAC sessions after orientation on the job. They are supervised by the health workers. The clients are supposed to be scheduled monthly to receive the sessions and at these sessions effort is made to understand the children’s drug administration, the barriers to adherence, social support, and opportunities to improve adherence using the 5 As (Assess, advise, assist, agree, arrange) [15]. All the information is recorded in the counselling notes and monitored and when the third session is done and the patient reports good adherence, a repeat viral load test is done.

Viral load tests are run at one central laboratory for the whole nation, the Central Pubic Health Laboratories (CPHL) in the capital city. The samples are drawn from the clients at the facility and sent to a laboratory hub in the district and thereafter to CPHL for analysis. The results return via the same route and should be available within two weeks of dispatch for delivery to clients at a subsequent clinic visit. If a client is currently on a second line regimen and has two detectable viral load test results, they are considered for a resistance test.

All these steps are routinely registered in MOH tools (the viral load register, the non-suppressed viral load register and in the client files), all of which were source documents for this evaluation.

### Procedures

A data abstraction form was used to collect data on the variables of interest from the source documents at all the 15 participating facilities. This was piloted and done by a group of nurses and data officers over a two week period. A list of the ART clinic numbers of children between 9 months and 19 years with a viral load above 1000 copies per ml was extracted from the viral load register at the facility. Using the ART clinic number, the clinical charts of these children were retrieved and reviewed to get additional information such as age, date of birth, date the child started ART, the current ART regimen, viral load test result and date of the result, whether or not the child had IAC, the dates the IAC sessions were held and the repeat viral load test result at the end of IAC. Additional information from the counselling notes on issues discussed with the children during the counselling sessions was also collected. Outcomes of those children who underwent all three IAC sessions were determined. The possible outcomes were; achievement of viral suppression, viral load remained unsuppressed and patient not switched, viral load remained unsuppressed and patient switched to second line, dead, transferred out and lost to follow up (if patient did not attend any recorded appointment in the preceding 12 weeks).

### Data management and analysis

The collected data was entered into a database and cleaned. Data extraction forms were reviewed daily for completeness. Analysis was done using stata statistical software version 13. Socio-demographic data was presented as frequencies and means, and IAC outcomes were presented as proportions and percentages of the children that were eligible for IAC. Dates to completion of the sessions was summarised using survival curves.

### Ethical consideration

Ethical approval for retrospective analysis and dissemination of results from routine program data was obtained from the the School of Health Sciences Research Ethics Committee at Makerere University College of Health Sciences (REC No: 2017–083). Approval from the Uganda National Council for Science and technology (SS 4538) was also obtained. Since this was a retrospective analysis of de-identified data a waiver of consent was also obtained from the same IRB.

## Results

### Socio-demographic characteristics of participants

Overall, there were 449 (16%) of all children in care who had a viral load above 1000 copies per ml (see Table 1). There was no significant difference in the sex of the children. The mean time on ART at the time that the child had a detectable viral load was 3.5 years (SD 5.8). The children who had been on ART for less than 3.5 years had a mean age of 8.7 years, compared to 10.8 years for the ones who had been on ART for more than 3.5 years (*p* value < 0.001). Majority of the children in both groups were in the 10 to less than 20 years age group 94 (39%) for those on ART less than 3.5 years and 98(51%) for those who had not been on ART for more than 3.5 years. Majority of the children were on a Nevirapine based regimen 160 (64.3%) in the group on ART less than 3.5 years and 160 (80.4%) in the older group. Most children 277 (62%) were living in urban areas.

**Table 1:** The demographic characteristics of the children with a detectable viral load

Characteristic	Number (percentage) *N* = 449		*P* values
	On ART < 3.5 yrs. at first detectable viral load (*N* = 249)	On ART > 3.5 yrs. at first detectable viral load (*N* = 200)	
Age (years) Mean, (SD)	8.7 (5.5)	10.8(4.4)	0.001
Age groups 17 missing			
< 3 years	39(16)	0	< 0.001
3- < 6 years.	70 (29)	29 (15)	
6–9 years	39(16)	63 (33)	
10–19 years	94(39)	98 (51)	
Sex			
Male	114 (45.8)	95 (47.5)	0.395
Female	135 (54.2)	105 (52.5)	
ART regimen 1 missing			
Efavirenz based	69 (27.7)	26 (13.1)	< 0.001
Nevirapine based	160 (64.3)	160 (80.4)	
PI based	20 (8)	13 (6.5)	
Area they are living			
Urban	155 (62.3)	122 (61)	0.431
Rural	94 (37.8)	78 (39)	

### Completion of IAC sessions and resultant outcomes

**Fig. 1:**
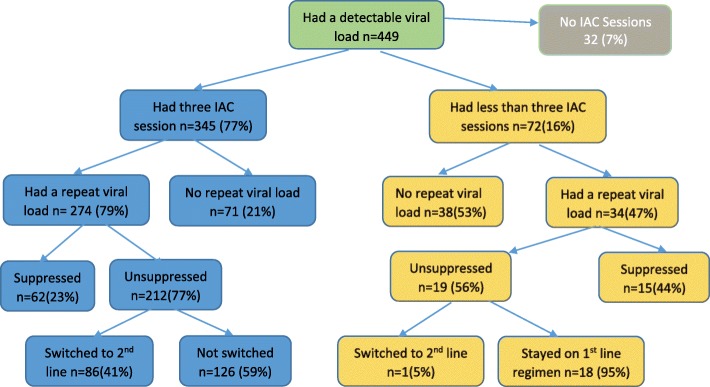
Showing actions taken for those that completed three IAC sessions compared to those who did not go through the three IAC sessions

Of all the 449 children with a viral load above 1000 copies per ml, 345 (77%) had all three IAC sessions within 400 days of receipt of VL result, 32(7%) did not receive any session and 72 (16%) received either one or two sessions. Of those that received all three sessions, 274/345 (79%) had a repeat viral load test, of whom 212/274 (77%) were unsuppressed and 62 (23%) were suppressed. Overall, of the children with unsuppressed viral load 86/212 (41%) were switched to a second line regimen and 126/212 (59%) remained on their first line regimen as adherence issues had not yet been addressed. 72/449 (16%) received less than 3 IAC sessions. Of these, 34/72 (47%) went ahead to get a repeat viral load test and 38/72 (53%) did not. 15/34 (44%) were suppressed on repeat viral load test and 19/34 (56%) were not suppressed. Of the children with unsuppressed viral load, 18/19 (95%) stayed on first line and 1/19 (5%) were switched to a second line regimen. See Fig. 1.

### Differences between children that were suppressed and those that weren’t after the IAC sessions

In general children that had HIV RNA viral suppression after three IAC sessions were not different from those that did not have HIV RNA viral suppression. There was no statistically significant difference in suppression rates across the different age groups, by clinic type (urban versus rural, hospital or lower facility) or by time to completion of the three IAC sessions. However, more children on a PI-based regimen achieved viral suppression, relative to those that were on non-PI containing regimen. See Table 2.

**Table 2:** The characteristics of children that had HIV viral load suppression and those that did not after receiving IAC sessions

Variables	Unsuppressed after 3 IAC sessions *n* = 206	Suppressed after 3 IAC sessions *n* = 65	*P* value
Duration on ART at failure	3.37 (2.27)	3.92 (2.96)	0.1160
Duration in days between result and completion of 3 IACs	227 (160)	192 (148)	0.118
Sex			
Female	99 (48.1)	28 (43.1)	0.536
Male	107 (51.9)	37 (56.9)	
Regimen			
EFV based	44 (21.4)	14 (21.5)	< 0.001
NVP based	154 (74.8)	40 (61.5)	
PI based	8 (3.9)	11 (16.9)	
Age			
< 3 years	20 (10.1)	4 (6.3)	0.175
3- < 6 years	47 (23.6)	11 (17.5)	
6–9 years	49 (24.6)	14 (22.2)	
10–14 years	51 (25.6)	26 (41.3)	
15–19 years	32 (16.1)	8 (12.7)	
Type of Clinic			
Urban	114 (74)	40 (26)	0.270
Rural	92 (79)	25 (21)	
Time to Completion of three IAC sessions			
Within 90 days	21 (21%)	77 (79%)	0.482
More than 90 days	44 (26%)	123 (74%)	

### Time to completion of IAC sessions

**Fig. 2:**
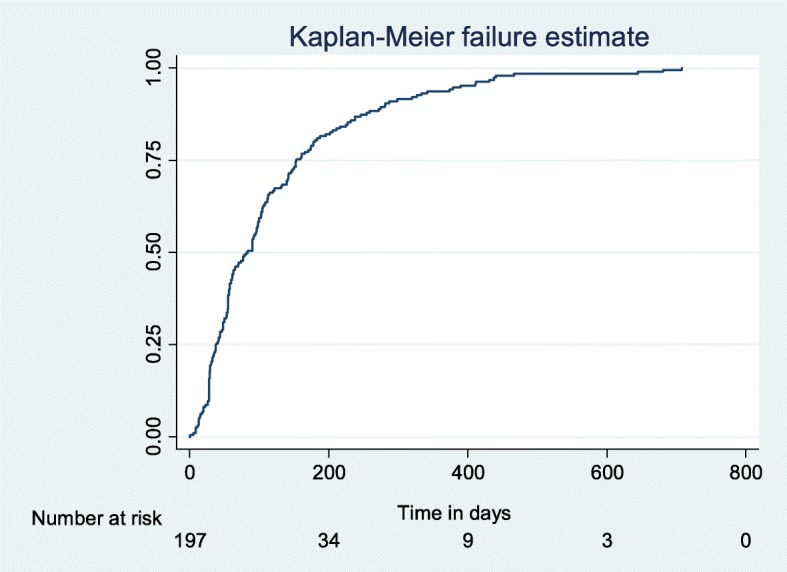
Time to 1st IAC session from date of detectable viral load

**Fig. 3:**
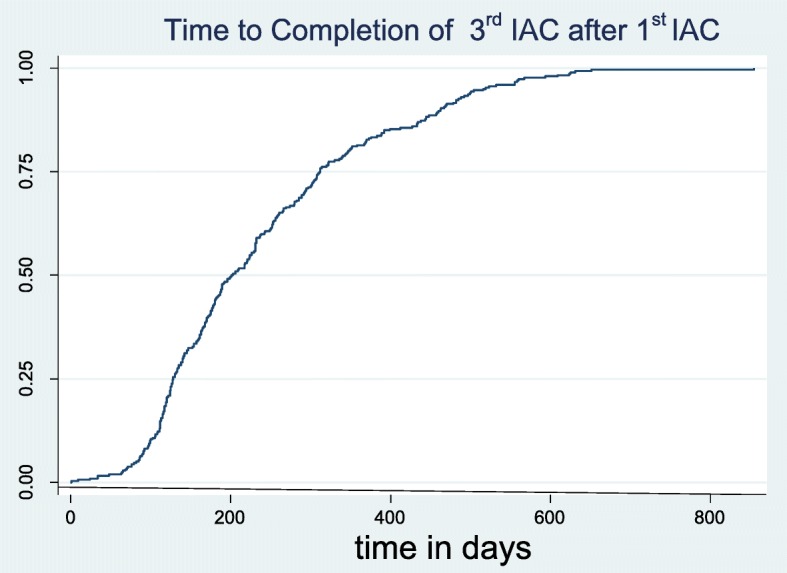
Showing the time in days to completion of 3rd IAC sessions after a detectable viral load result

**Fig. 4:**
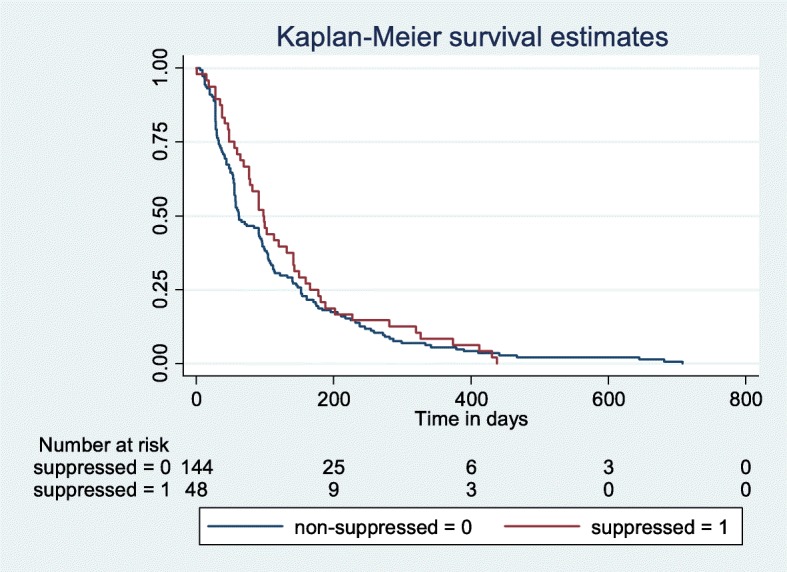
Showing time from the 3rd IAC Session to repeat viral load by those who are suppressed compared to those who are not suppressed

An analysis of the timeliness of receiving the IAC sessions was made. Figure 2 shows that 75% of the children received the first IAC session up to 200 days after the detectable viral load test result was available at CPHL. Fig. 3 shows that 75% completed the three IAC sessions after 350 days. Figure 4 shows that 75% of the children had a repeat viral load test done within 180 days of finishing the three IAC sessions. The graph also shows that those who are suppressed get a repeat viral load test much earlier than those who are unsuppressed.

## Discussion

In this cohort, we report that IAC sessions were completed for 77% of children identified with viral non-suppression; that 16% had less than three IAC sessions and another 7% did not receive any sessions within the year. Only 23% of the patients that had IAC and a repeat viral load attained viral suppression; We postulate that IAC did not produce the expected high proportions of viral suppression (above 70%) probably due to barriers at facility level or at patient level that need further exploration. At the health facility; the time taken to conduct the counselling session might not be sufficient because of the patient load, the person delivering the message might also affect the message particularly the level of education and working experience. At patient level, there are multiple psychosocial factors that affect adherence which if not addressed could lead to the observed ineffectiveness of counselling. These include non-disclosure of status, stigma, school related activities, dependence on a caregiver, having a non-biological caretaker and forgetfulness [9, 17–19].

A study found that treatment fatigue and depression among care takers directly affected adherence and subsequently viral suppression [20]. This is important because children and adolescents depend for the most part on their caregivers to provide ART and to bring them to their appointments [8]. In Nigeria, it was found that caretakers who are supported through psychosocial support have children who achieve better viral suppression [21]. It has also been found that children with the most psychosocial factors also have the worst adherence to ART [22]. In this program, many of the younger children are given adherence support through their caregivers but this was not observed to make a difference for these children. Therefore, there is need to understand the dynamics and family characteristics that affect adherence in children for improved outcomes.

It is also possible that the barriers that were addressed during the counselling sessions were not the real ones since adolescents have been known to have unique challenges that prevent them from staying adherent [23]. However, it could just be due to the fact that children sometimes have detectable viral loads in the absence of resistance [24]. Two studies have found that adolescents are less likely to achieve complete viral suppression compared to adults [25, 26]. Children that participate in leisure activities and also receive psychosocial support are more likely to be retained in care and also have better viral suppression [21]. Although these studies were not conducted on Ugandan children, their findings could still be applicable to this population. In Kenya and Uganda, a study done among children newly initiating ART showed that if barriers such as long waiting time and proper understanding of need for ART are addressed, children achieve viral suppression [27]. There is a need to further understand specific barriers among the children that did not attain viral suppression after 3 IAC sessions, particularly if they did not have drug resistance.

It was found that of the 212 (77%) that did not suppress the HIV virus after IAC, only 41% were switched to a second-line ART regimen while the remaining 59% continued receiving a non-effective ART regimen. This is unfortunate as children with high viral loads have been shown to stay on failing regimen for long periods of time [10]. Our findings are consistent with those of a study done in Swaziland where children who received enhanced adherence counselling from a lay counsellor had a re-suppression rate of 36%. Children and adolescents below 20 years were also more likely not to achieve suppression compared to adults, the mean age on ART for this group was 2.7 years [28]. In Rwanda, it was found that children are also more likely to continue having high viral loads despite routine adherence support. These children had similar ages and had also been on ART for a mean 3.4 years with 71% on a Nevirapine based regimen [2]. These findings are different from a study in South Africa that found that children with adherence support achieve higher suppression rates [16]. However, in this South African study, the adherence support was provided right at the start of ART and it was mostly provided in the community. There is need for paediatric ART programs to consider intensified adherence counselling right from the start of ART and how it can be implemented in a program setting to improve the HIV viral suppression levels.

IAC sessions could appear not to be effective as the unsuppressed children could have been receiving a failing ART regimen with undiagnosed drug resistance as resistance profiles are not routinely conducted [15].

The IAC sessions were not conducted according to the recommendation of having three sessions each one month apart and then repeating the viral load. The recommended time to complete is 90 days and yet 50% of the participants had received their IAC sessions by 200 days. Although this did not appear to make a difference for this cohort.

We reported no significant differences among children that attained and those that did not attain viral suppression after 3 IAC sessions, except higher use of PI-based regimen among those that suppressed the virus. These findings are comparable with reports from South Africa that children on a boosted PI regimen achieved viral suppression even with suboptimal adherence [29].

### Study limitations

One of the national program limitations is that resistance testing is reserved only for those failing on a second line regimen and was not done for any of the children in this cohort. Routine monitoring for viral load was newly introduced in the country in late 2015 and many of the children were accessing the viral load test for the first time; they could have been failing on their regimens long before the test and this might have reduced their chances of achieving viral suppression. The study utilized retrospective data that is routinely collected at public health facilities and the researchers had no control over this in either collection or quality assurance. All effort was made to verify the data and ensure it is of a comparatively good quality for analysis. Data on the content and quality of the counselling sessions was not available. We therefore recommend monitoring and evaluation of the quality of the counselling offered in IAC sessions, which is likely to affect IAC outcomes. Similarly, information on other factors that affect adherence such as disclosure status was not available in the records.

Improved adherence after the IAC sessions was measured using self-report for the adolescents and caregiver reports for the younger children, which might not be very objective. However, a study done in Swaziland found that self-report can be used to effectively report on adherence among adolescents [30]. Clearly more needs to be done to effectively address individual and facility level factors that affect ART adherence and viral suppression including biological factors such as resistance among children and adolescents under IAC.

## Conclusion

HIV-RNA viral suppression after 3 sessions of IAC was 23% and up to 50% of patient completed IAC within 200 days instead of the recommended 90 days. Adherence to the recommendations on how to handle clients that have unsuppressed viral loads was very low. As we move towards having 90% of ART-treated children and adolescents achieve and maintain viral suppression, there is need to re-evaluate the implementation of IAC among children and adolescents and to consider resistance testing for those with multiple detectable viral loads.

## Abbreviations

ART: Anti-retroviral Therapy
CPHL: Central Public Health Laboratories
HIV: Human Immunodeficiency Virus
IAC: Intensified Adherence Counselling
IDI: Infectious Diseases Institute
PI: Protease Inhibitor
RNA: Ribonucleic Acid
SD: Standard Deviation
UNAIDS: United Nations AIDS
WHO: World Health Organisation
